# Persistent contamination of a duodenoscope working channel in a non-clinical simulated ERCP setting

**DOI:** 10.1055/a-1814-4379

**Published:** 2022-05-05

**Authors:** Judith A. Kwakman, Michiel L. Bexkens, Marco J. Bruno, Margreet C. Vos

**Affiliations:** 1 Department of Gastroenterology and Hepatology, Erasmus Medical Center, Rotterdam, The Netherlands; 2 Department of Medical Microbiology and Infectious Diseases, Erasmus Medical Center, Rotterdam, The Netherlands

## Abstract

**Background** To mitigate duodenoscope contamination, recent design enhancements have primarily focused on the distal tip. However, the working channels remain unchanged, which may be linked to biofilm formation. We assessed the persistence of microorganisms, indicative of biofilm formation, in reprocessed duodenoscopes in a non-clinical endoscopic retrograde cholangiopancreatography (ERCP) simulation setting.

**Methods** Three new duodenoscopes were over-soiled in non-clinical ERCP simulations followed by reprocessing. After 40 tests, the *Pseudomonas aeruginosa* strain in the soil (Pa-type 1) was switched to a different *P. aeruginosa* strain (Pa-type 2) for 20 subsequent tests. Cultures of the tip and working channel were acquired after high level disinfection and overnight storage.

**Results** One duodenoscope showed persistent growth of *P. aeruginosa* from the fifth test until the end of the study. Pa-type 1 remained present until the end of the study in the cultures of this duodenoscope, even after discontinuation of exposure to that specific strain. The other two duodenoscopes only showed incidental contamination.

**Conclusion** Persistent contamination by Pa-type 1 was seen in one out of three duodenoscopes after exposure to supraphysiological levels of gut microorganisms. No clear explanation was found for this persistent contamination as exposure and handling were identical and no abnormalities of this particular duodenoscope were identified by borescope inspection.

## Graphical abstract

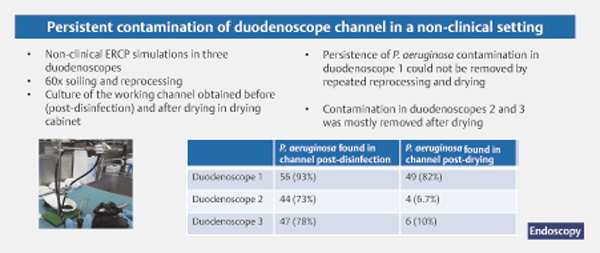

## Introduction

There has been increasing attention toward biofilm as the source of persistent contamination of duodenoscopes. A biofilm is an accumulation of microorganisms on a surface enclosed in a matrix of exopolysaccharides. The existence of microorganisms in a biofilm protects them from outside influences, such as detergents and antibiotics 1 2 .

Research on duodenoscope biofilms has so far mainly focused on existing biofilms in clinically used endoscopes and on in vitro biofilms in simulation channels 3 4 . In this study, we examined the persistence of microorganisms in the distal tip and working channels after exposure to supraphysiological loads of gut microorganisms in a non-clinical ERCP simulation setting.

## Methods

### Study design

Three brand new duodenoscopes (all DEC ED34-i10T2; Pentax Medical, Dodewaard, The Netherlands) were inoculated with an artificial test soil (ATS) whose composition and viscosity resembles gastrointestinal secretions 5 . The ATS (ATS2015; Healthmark Industries Company Inc., Fraser, Michigan, USA) was inoculated with four microorganisms together ( *Pseudomonas aeruginosa* [ATCC 27853], *Klebsiella pneumoniae* [ATCC 13883], *Enterococcus faecium* [ATCC 35667], *and Escherichia coli* [ATCC 25922]) in a supraphysiological concentration of 10 ^8^ colony forming units (CFU)/mL of each strain (compared with the average of 10 ^7^ CFU/mL normally found on used duodenoscopes 6 ).

In phase 1, the three duodenoscopes underwent 40 tests each, followed by 20 tests in phase 2 in which *P. aeruginosa* (ATCC 27853) was replaced with a different *P. aeruginosa* strain (ATCC 15442), referred to as Pa-type 1 and Pa-type 2, respectively.

### Soiling

The duodenoscopes were placed in a tripod in an S curve. A sterilized reusable biopsy forceps was introduced 10 times into the working channel immediately after the distal tip was placed in a container with 50 mL of ATS. The ATS was produced in batches of 500 mL and was used within a few days (maximum 1 week). Each time the biopsy forceps was in situ, the forceps elevator was moved up and down five times and alternatingly the suction and flush valves were pressed five times. Both valves were pressed for a few seconds, until soil was seen within the suction connection tube or water came out of the distal tip. The complete soiling procedure took approximately 10 minutes.

Bedside cleaning was immediately performed according to the manufacturer’s instructions for use. After 15 minutes, the duodenoscopes were reprocessed according to the instructions for use, including manual cleaning, immediately followed by automated high level disinfection (HLD; Wassenburg WD440 PT; Wassenburg Medical, Dodenwerf, The Netherlands) and storage in a drying cabinet (Wassenburg DRY300 D, Wassenburg Medical). The detergent used was Mediclean Forte and the disinfectant was Neodisher Septo PAC (both Dr. Weigert, Hamburg, Germany).

### Sampling

Directly after HLD and again after overnight storage, two samples were taken for culture. A sample of the distal tip was collected using a flocked eSwab with accompanying Amies fluid (eSwab; Copan, Brescia, Italy) 7 . A flush-brush-flush sample of the suction/working channel was collected using two flushes of 20 mL sterile water and a disposable brush (CS5522A; Pentax). Then 40 mL of neutralizer (Dey-Engley broth; Merck KGaA, Darmstadt, Germany) was added to the channel sample 8 and 1 mL to the eSwab sample. After it had been vortexed, the 2-mL eSwab medium was poured onto tryptic soy agar plates. The flush samples were filtered through a 0.45-µm filter, which was placed on R2A agar. Samples were incubated at 35 °C and examined for growth after 72 hours.

Culture results were presented in CFUs per microorganism per sample site, with a maximum of 100 CFUs per sample. To differentiate between growth of Pa-type 1 and Pa-type 2 in phase 2, matrix-assisted laser desorption time of flight mass spectrometry (MALDI-TOF MS) combined with cluster analysis was used ( **Appendix 1 s** , see online-only Supplementary material).

### Outcomes

The presence of applied bacterial species was assessed per culture. The secondary outcome was persistence of Pa-type 1 in cultures acquired in phase 2, despite the duodenoscopes no longer being exposed to this contaminant, indicating the existence of biofilm. At baseline and after every 10 tests, the working channels of the duodenoscopes were inspected for abnormalities using a borescope (Flexible Inspection Scope FIS-005; Healthmark Industries Company Inc.).

Data collection was performed using SPSS, version 25 (2016; IBM Corp., Armonk, New York, USA). The proportions of positive culture outcomes for each of the three duodenoscopes are provided with their 95 %CIs. *P* < 0.05 was considered a statistically significant difference between the duodenoscopes.

## Results

Per duodenoscope, 60 tests were performed with two sampling moments per test, resulting in 120 samples of both the tip and working channel per duodenoscope. Post-HLD, 10 samples of the tip (5.6 %) were positive for any of the applied microorganisms and two samples were positive post-drying (1.1 %) ( Table 1 ). All 10 positive post-HLD samples of the distal tip (100 %) were followed by a negative sample post-drying. Channel samples were positive 161 times post-HLD (89.4 %) and 60 times post-drying (33.3 %). Of the 161 positive post-HLD samples, 107 (66.5 %) were followed by a negative sample post-drying.

**Table 1:** Number of positive cultures for the four indicator microorganisms per duodenoscope (n = 60 for all acquired samples).

	*P. aeruginosa* , n (%)	*K. pneumonia* , n (%)	*E. coli* , n (%)	*E. faecium* , n (%)	Any indicator microorganism, n (%)
**Duodenoscope 1**					
Tip after HLD	3 (5.0 %)	1 (1.7 %)	1 (1.7 %)	0	3 (5.0 %)
Tip after drying	1 (1.7 %)	0	0	0	1 (1.7 %)
Channel after HLD	56 (93.3 %)	24 (40.0 %)	9 (15.0 %)	0	57 (95.0 %)
Channel after drying	49 (81.7 %)	3 (5.0 %)	1 (1.7 %)	0	49 (81.7 %)
**Duodenoscope 2**					
Tip after HLD	3 (5.0 %)	1 (1.7 %)	1 (1.7 %)	0	3 (5.0 %)
Tip after drying	0	0	0	0	0
Channel after HLD	44 (73.3 %)	28 (46.7 %)	21 (35.0 %)	10 (16.7 %)	53 (88.3 %)
Channel after drying	4 (6.7 %)	0	0	0	4 (6.7 %)
**Duodenoscope 3**					
Tip after HLD	2 (3.3 %)	2 (3.3 %)	0	1 (1.7 %)	4 (6.7 %)
Tip after drying	0	0	0	1 (1.7 %)	1 (1.7 %)
Channel after HLD	47 (78.3 %)	25 (41.7 %)	26 (43.3 %)	2 (3.3 %)	51 (85.0 %)
Channel after drying	6 (10.0 %)	0	1 (1.7 %)	0	7 (11.7 %)

HLD, high level disinfection.

Over the whole study period, only in the channel samples collected post-drying was a significant difference found between duodenoscope 1 and duodenoscopes 2 and 3, with 49 positive samples (81.7 %, 95 %CI 70.0 %-90.5 %) compared with 4 (6.7 %, 95 %CI 1.9 %-16.2 %) and 7 positive samples (11.7 %, 95 %CI 4.8 %-22.6 %), respectively.

### Duodenoscope 1

**Fig. 1:**
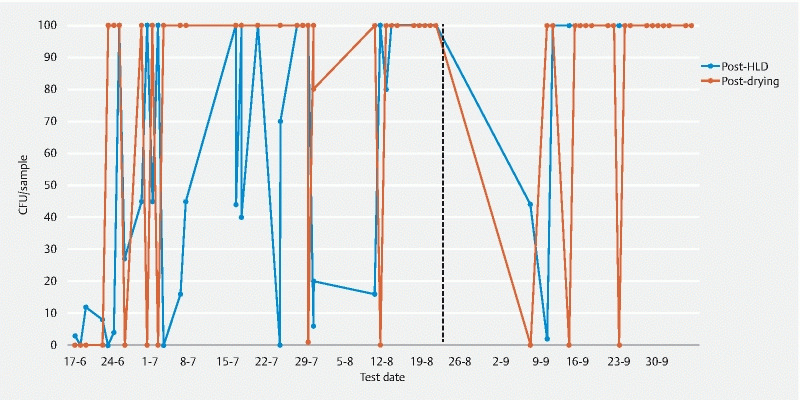
Growth of *Pseudomonas aeruginosa* in the channel samples of duodenoscope 1 after high level disinfection (HLD) and drying. In phase 2, all post-HLD tests except test number 42 (test date 10–9) were found to be positive for both Pa-types 1 and 2. In test number 42, the culture showed only Pa-type 2. Of the post-drying tests, all but two of the positive samples were found to contain both Pa-types 1 and 2. In test number 42 (date 10–9), the culture showed only Pa-type 2. The sample of number 47 (date 17–9) was not typed. The dashed line indicates the start of phase 2. CFU, colony forming unit.

The tip of duodenoscope 1 was positive in 4 /120 cultures, three post-HLD and one post-drying ( Table 1 ). The channel samples were positive 24 times for *K. pneumoniae* post-HLD (40 %) and three times post-drying (5 %). Nine channel samples were positive for *E. coli* post-HLD (15 %) and one post-drying (1.7 %). No channel samples were positive for *E. faecium.* The channel cultures were positive 56 times for *P. aeruginosa* post-HLD (93.3 %) and 49 times post-drying (81.7 %) ( Fig. 1 ).

In phase 2, all except one post-HLD channel sample grew both Pa-types 1 and 2 *.* Three of the post-drying channel samples were negative for any growth of *P. aeruginosa.* In 15 of the 17 positive post-drying channel samples, both Pa-types 1 and 2 could be identified. Pa-type 1 was not found in post-drying test numbers 42 and 47.

### Duodenoscope 2

**Fig. 2:**
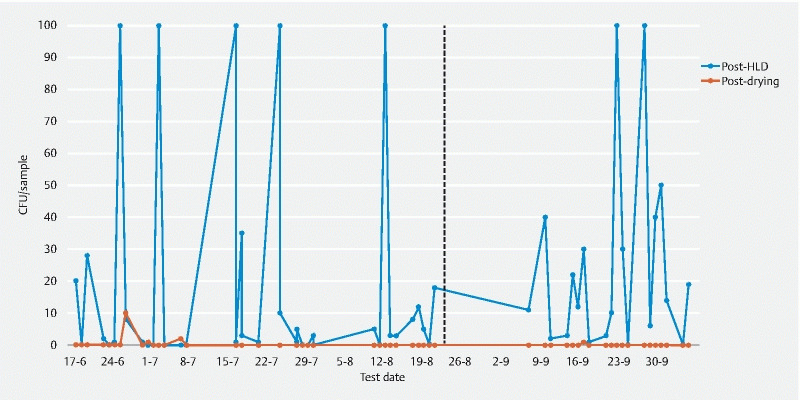
Growth of *Pseudomonas aeruginosa* in the channel samples of duodenoscope 2 after high level disinfection (HLD) and drying. In phase 2, all colonies found after HLD were Pa-type 2, the one colony found after drying was Pa-type 1. The dashed line indicates the start of phase 2. CFU, colony forming unit.

In duodenoscope 2, the distal tip was positive in three post-HLD samples and negative in all post-drying samples. In the post-HLD channel samples, *K. pneumoniae* was found 28 times (46.7 %), *E. coli* 21 times (35.0 %), and *E. faecium* 10 times (16.7 %). These three microorganisms were not cultured in any post-drying channel samples. Growth of *P. aeruginosa* was found in 44 post-HLD channel samples (73.3 %), but only in four samples post-drying (6.7 %) ( Fig. 2 ). In phase 2, the single CFU of *P. aeruginosa* found post-drying was identified as Pa-type 1. The positive post-HLD cultures in phase 2 harbored only Pa-type 2.

### Duodenoscope 3

**Fig. 3:**
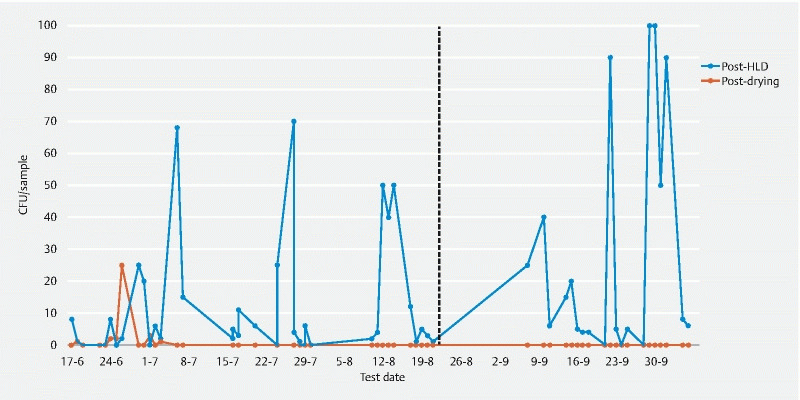
Growth of *Pseudomonas aeruginosa* in the channel samples of duodenoscope 3 after high level disinfection (HLD) and drying. In phase 2, any positive cultures showed growth of Pa-type 2 only. The dashed line indicates the start of phase 2. CFU, colony forming unit.

The distal tip of duodenoscope 3 was cultured positive four times post-HLD and once post-drying. *K. pneumoniae* was found in 25 post-HLD channel samples (41.7 %), but never post-drying. *E. coli* was found in 26 post-HLD samples (43.3 %) and one post-drying sample (1.7 %). *E. faecium* was found twice post-HLD (3.3 %) and never after drying. *P. aeruginosa* was found in 47 post-HLD channel samples (78.3 %) and in six post-drying samples (10.0 %) ( Fig. 3 ). All *P. aeruginosa* grown in phase 2 were Pa-type 2.

### Channel inspection

Seven borescope inspections were performed per endoscope, at baseline and after every 10 tests. Apart from superficial scratches at the entrance of all three working channels, no abnormalities were visualized in any of the working channels.

## Discussion

This is the first study demonstrating the persistence of *P. aeruginosa* in a non-clinical ERCP simulation setting, as shown by the presence of *P. aeruginosa* in cultures of one duodenoscope after discontinuing exposure to this specific microorganism. This is suggestive of the formation of a biofilm inside this duodenoscope. The results of this experimental study show the persistent nature of the contamination in one duodenoscope, which existed over a period of 3 months despite repeated reprocessing and drying according to the manufacturer’s instructions on a daily basis. No predisposing factor could be identified to explain why only one of the three identical duodenoscopes was affected.

The results of this study confirm the importance of endoscope drying. Many post-HLD cultures of duodenoscopes 2 and 3 were positive for growth of indicator microorganisms. But, after overnight storage in a drying cabinet, almost all samples collected from these two duodenoscopes were negative. Microorganisms can reside in droplets in the channels and then develop biofilms in these moist environments. Currently, many guidelines allow the use of wet duodenoscopes where the next ERCP is within a certain period (4 hours in the Dutch guideline 7 ). However, our results indicate that, right after HLD, microorganisms may still be present, which could easily be removed by drying, provided no biofilm has yet developed. This calls for a revision of, or at least further investigation of, the use of wet endoscopes.

This experiment was continued without acting upon the culture results as would have happened in clinical practice. National and international guidelines advise the quarantining of endoscopes found to carry gut microorganisms such as *P. aeruginosa* . Depending on the advised intervals for surveillance culture, it is more or less plausible that contamination of a duodenoscope with *P. aeruginosa* can go unnoticed for such a long period. In the Dutch situation, where surveillance is recommended every 6 months 9 , it is possible that contaminated duodenoscopes are used for months before this is detected. This could be prevented by more regular surveillance measurements.

Currently, the focus on improving the design to reduce duodenoscope contamination is mainly directed toward the distal tip and forceps elevator. This is demonstrated by the newest duodenoscope models equipped with disposable distal tips and the recent US Food and Drug Administration (FDA) advice to move away from duodenoscopes with fixed endcaps 10 . However, channel contamination might still occur in these newly designed duodenoscopes. The ultimate solution could be to use disposable single-use duodenoscopes. Unfortunately, particularly costs and potentially environmental impact currently prohibit a collective switch to disposable duodenoscopes.

A few limitations of this study hamper its generalization to the clinical situation. First, only a single duodenoscope model was used. This model was chosen because it is often used and its disposable cap is recommended by the FDA 10 . Indeed, we did not see contamination in the distal tip, but only in the channel. In other studies, it has been shown that biofilm formation is possible in other duodenoscope models as well 11 12 . Second, the ERCP simulation is not completely comparable to clinical ERCPs. In this study, only a biopsy forceps was used in the working channels. In clinical ERCP procedures, a variety of instruments are used, which might cause more wear and tear of the working channel. Also, the duration of most ERCP procedures in patients exceeds the 10 minutes of our simulated ERCP procedures.

The soil used in this study contained supraphysiological loads of four types of pathological gut microorganisms, it is unlikely that duodenoscopes are exposed to this kind of contamination on a regular basis. In the upper gastrointestinal tract, a more diverse flora can be found which might less often lead to a biofilm of one specific microorganism in exposed endoscopes. Also, the absence of bile in the ATS might be beneficial for the survival of the four applied microorganisms. Additional studies are needed to validate these findings and to further examine biofilm formation in the working channel, under routine clinical conditions. Also, it would be interesting to investigate biofilm formation in other types of endoscopes, such as gastroscopes and colonoscopes, which have a slightly different design and have been less often involved in outbreaks.

In conclusion, in this non-clinical ERCP simulation study, duodenoscopes were exposed to supraphysiological loads of pathological gut microorganisms during 60 cycles. This led to the persistence of Pa-type 1 in the working channel of one out of three duodenoscopes, despite continued disinfection cycles and even after stopping exposure to this specific Pa-type 1. This finding is highly suggestive of biofilm formation; however, this was not visualized by microscopic assessment. Our results indicate that future research and development should focus more on the working channel to continue to reduce the risk of biofilm formation.

## Supplementary material

Supplementary material Zusatzmaterial
