# Supplementary material for: Persistent contamination of a duodenoscope working channel in a non-clinical simulated ERCP setting
Source: Endoscopy. 2022 May 5;54(11):1085–90. doi: 10.1055/a-1814-4379 (PMC9613439; doi:10.1055/a-1814-4379)
Supplement: Supplementary file 1 — Supplementary material [file 21193supmat_10-1055-a-1814-4379.pdf]

Supplementary material

Assessment of biofilm formation inside a duodenoscope working channel in a non-clinical simulated ERCP setting

Judith A. Kwakman, Michiel L. Bexkens, Marco J. Bruno, Margreet C. Vos

Appendix 1s. Description of MALDI-TOF MS differentiation method Pa-Type 1 and 2.

Introduction

To differentiate between growth of Pa-type 1 and Pa-type 2 in phase 2, Matrix Assisted Laser Desorption Time of Flight Mass Spectrometry (MALDI-TOF MS) combined with cluster analysis was used. MALDI-TOF MS can be used for the typing of bacteria and is regarded as the golden standard in clinical microbiology laboratories (1). Identification of bacteria is done by comparing spectra generated by the MALDI-TOF MS device, to a database of known reference spectra, thereby providing species identification. This technique is mainly used for species typing, but fingerprinting or subspecies identification is also possible (2, 3). To do so, small but consistent differences between spectra of closely related species are analyzed and compared to reference strains. Here we have used this technique to distinguish *P. aeruginosa* ATCC 27853 (Pa-Type 1) from *P. aeruginosa* ATCC 15422 (Pa-Type 2).

Methods

Reference spectra were generated for both Pa-type 1 and Pa-type 2 via the following procedure. Eight samples of each strain were subjected to an extended extraction (4) after which 1 µl extract was spotted on the MALDI-target in triplicate and overlaid with 1 µl matrix (alpha-cyano-4-hydroxycinnamic acid, CHCA), resulting in a total of 24 recorded spectra per strain. Spectra were recorded in the 2000-20000

Supplementary material

m/z (mass over charge) range. A calibration with a Bacterial test standard (BTS) was performed prior to all MALDI-TOF MS analysis, and all strains were identified as *P. aeruginosa* with a log-score >2 using the Biotyper platform of Bruker (Bruker Daltonics, Mannheim,Germany). The resulting raw FID-spectra were converted to mzML using Compassxport (Software version 3.0.9.2, Bruker Daltonics) and imported in the Bionumerics Software package (Version 7.6.3, Applied Maths, St. Martens-Latem, Belgium) as spectrum type data, mzML data.

Preprocessing on the spectra was performed via predefined templates in Bionumerics, including resampling, smoothing and baseline correction followed by peak detection with a 10:1 signal to noise ratio. Unique discriminatory peaks were selected via a Mann-Whitney *U* test, comparing presence and intensity of peaks detected in the reference spectra of Pa-Type 1 and Pa-Type 2. The selected discriminatory peaks were used for typing of the unknown samples via similarity cluster analysis.

The typing results were then analysed for accuracy using a Jackknife resampling method.

When growth of *P. aeruginosa* was found in any culture, a maximum of ten CFU, randomly selected, were subjected to this MALDI-TOF MS analysis to differentiate between Pa-Type 1 and 2. If only one of the two types was found, more CFU were subjected to this analysis to avoid missing the other type due to the selection of only ten CFU.

Results

From the analysis of Pa-Type 1 (24 spectra) and Pa-Type 2 (24 spectra) seven statistically significant peaks were selected. These peaks were present in the mass-spectrum at 2723.25, 3664.21, **7312.06**, 12142.68, 3455.34, **6912.05**, **7074.25** m/z. (**Bold** values are further shown in figure 1s). Cluster analysis using the peaks identified 145 samples as Pa-type 1 and 502 samples as Pa-type 2. Jackknife resampling analysis showed that typing of these strains as either type-1 or type 2 was >99% successful.

Supplementary material

Of all positive P. aeruginosa cultures in phase 2, a total of 679 distinct CFU were subjected to MALDI-TOF MS analysis. Of these 679 CFU, 634 (93.4%) could be identified as either Pa-Type 1 or 2 in the first analysis. Thirteen CFU coming from nine different cultures were subjected to MALDI-TOF MS a second time, because they could not be identified and could then all be successfully identified as either one of the types. Of the other 32 CFU that could not be identified the first time, other CFU of the same original culture already showed presence of both Pa-Type 1 and 2, therefore, these CFU were not analyzed a second time.

Figure 1s. Mass-spectrum overlay of ATCC 27583 (black) and ATCC 15422 (orange).
Mass over charge (m/z) is shown on the x-axis, y-axis describes peak intensity in arbitrary units (a.u.)
Shown is a section of one spectrum each (out of 24) for ATCC 27853 (Pa-Type 1) and ATCC 15422 (Pa-Type 2), region shown is from ~6900 m/z until ~7400 m/z. Black arrows indicate a difference in peak intensity, arrow number 1 is placed at **6912** m/z**,** arrow number 2 is placed **7074** m/z and arrow number 3 is placed **7312** m/z.
The presence or absence of these three peaks, along with the peaks at 2723.25, 3664.21, 12142.68 and 3455.34 m/z allowed for clear distinction between Pa-type-1 and Pa-type-2.

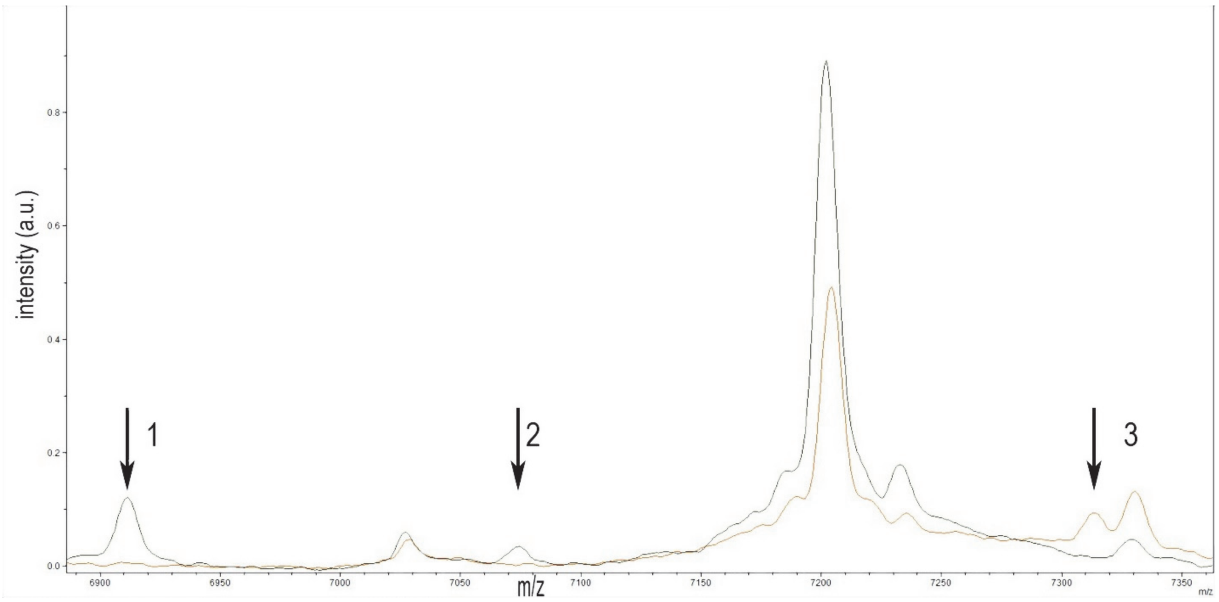

Supplementary material

References

1. van Belkum A, Welker M, Pincus D, Charrier JP, Girard V. Matrix-Assisted Laser Desorption Ionization Time-of-Flight Mass Spectrometry in Clinical Microbiology: What Are the Current Issues? Ann Lab Med. 2017;37(6):475-83.
2. Nagy E, Urbán E, Becker S, Kostrzewa M, Vörös A, Hunyadkürti J, et al. MALDI-TOF MS fingerprinting facilitates rapid discrimination of phylotypes I, II and III of Propionibacterium acnes. Anaerobe. 2013;20:20-6.
3. Pérez-Sancho M, Vela AI, Horcajo P, Ugarte-Ruiz M, Domínguez L, Fernández-Garayzábal JF, et al. Rapid differentiation of Staphylococcus aureus subspecies based on MALDI-TOF MS profiles. J Vet Diagn Invest. 2018;30(6):813-20.
4. Alatoom AA, Cunningham SA, Ihde SM, Mandrekar J, Patel R. Comparison of direct colony method versus extraction method for identification of gram-positive cocci by use of Bruker Biotyper matrix-assisted laser desorption ionization-time of flight mass spectrometry. J Clin Microbiol. 2011;49(8):2868-73.
